# Molecular epidemiology and antimicrobial resistance in *Clostridioides difficile* strains isolated from children and adolescents in a tertiary referral pediatric hospital in Fortaleza, Brazil

**DOI:** 10.1016/j.bjid.2024.103767

**Published:** 2024-06-03

**Authors:** Hildenia Baltasar Ribeiro Nogueira, Cecília Leite Costa, Carlos Quesada-Gómez, Dvison de Melo Pacífico, Eliane de Oliveira Ferreira, Renata Ferreira de Carvalho Leitão, Gerly Anne de Castro Brito

**Affiliations:** a Hospital Infantil Albert Sabin, Fortaleza, CE, Brazil; b Universidade Federal do Ceará, Núcleo de Microscopia e Processamento de Imagens (NEMPI), Departamento de Morfologia Fortaleza, CE, Brazil; c Universidade de Fortaleza, Fortaleza, CE, Brazil; d Centro Universitário Christus, Fortaleza, CE, Brazil; e Facultad de Microbiología and Centro de Investigación en Enfermedades Tropicales, Universidad de Costa Rica, San José, Costa Rica; f Universidade Federal do Rio de Janeiro, Instituto de Microbiologia Paulo de Góes, Laboratório de Biologia de Anaeróbios, Rio de Janeiro, RJ, Brazil

**Keywords:** *Clostridioides difficile* infection, *C. difficile*-associated diarrhea, Children, Pediatric diarrhea

## Abstract

**Background:**

*C. difficile* has been increasingly reported as a cause of gastrointestinal disease in children, ranging from mild self-limiting diarrhea to severe conditions such as pseudomembranous colitis and toxic megacolon. Only two pediatric research groups reported the presence of *C. difficile* infection in Brazilian children, but no previous research has examined *C. difficile* infection among children in northeastern Brazil. This prospective cross-sectional study investigated the molecular epidemiology and antimicrobial resistance of *C. difficile* strains isolated from children and adolescents with diarrhea referred to a tertiary pediatric hospital in Brazil while exploring the associated risk factors.

**Results:**

Toxin positivity or *C. difficile* isolation was found in 30.4 % (17/56) samples. *C. difficile* was isolated from 35 % (6/17) samples. Four toxigenic strains were identified (tpi+, tcdA+, tcdB+, cdtB-, without tcdC deletions) belonging to PCR ribotypes and PFGE-pulsotypes: 046 (new pulsotype 1174), 106 (NAP11), 002 (new pulsotype 1274), 012 (new pulsotype NML-1235). Two of the six isolates belonging to ribotypes 143 and 133 were non-toxigenic. All toxigenic strains were sensitive to metronidazole and vancomycin. Regarding the clinical manifestation, diarrhea lasted an average of 11 days, ranging from 3 to 50 days and was often associated with mucus and/or blood. All six patients from whom the *C. difficile* was isolated had a chronic disease diagnosis, with these comorbidities as the main risk factors.

**Conclusion:**

Our study enhances our understanding of the present epidemiological landscape of *C. difficile*-associated diarrhea (CDI) among children in northeastern Brazil, reveling a substantial CDI frequency of 30.4 %, with toxigenic strains detected in 76.4 % of cases, highlighting a higher prevalence compared to earlier Brazilian studies. In the globalized world, an understanding of disease-generating strains, the associated risk factors, clinical manifestation, and antimicrobial sensitivity has fundamental epidemiological importance and draws attention to preventive measures, allowing for more decisive action.

## Introduction

*Clostridioides difficile*, a Gram-positive, spore-forming, anaerobic bacterium, is the foremost cause of healthcare-associated diarrhea worldwide. While it remains the primary contributor to healthcare-related diarrhea among adults in the United States, it is also increasingly acknowledged as a significant pathogen impacting children.^1^

The carriage rate of *C. difficile* is notably high among newborns, implying a commensal status for this bacterium within this specific population segment.^1^^,^^2^ For reasons still unknown, these babies colonized with toxigenic *C. difficile* rarely develop clinical symptoms, unlike older children and adults who are susceptible to severe diarrhea and colitis. Colonization rates tend to decrease with increasing age, with children older than two years exhibiting rates similar to those observed in adults.^2^ Nevertheless, toxigenic strains may also colonize asymptomatic infants during hospitalization and within the community.^2^

In recent decades, a significant increase in the incidence of CDI has been reported, even in populations previously considered low-risk, such as children. Despite the rising number of CDI cases in children, along with an increase in morbidity related to frequent recurrences,^3^ and evidence that infection in hospitalized children has a similar economic impact to that observed in adults,^4^ research on pediatric CDI remains considerably limited when compared to study in adults. Moreover, there has been a noteworthy rise in the prevalence rates of both Healthcare-Associated (HA-CDI) and Community-Associated (CA-CDI) CDI among pediatric populations.^5^, ^6^, ^7^

Given the limited information and diagnosis of *C. difficile* infection in Brazilian hospitals, especially in the Northeast region, and the global spread of this infection, our study aimed to conduct a local epidemiological investigation. Specifically, this study sought to identify comorbidities and risk factors associated with CDI in pediatric patients and investigate molecular epidemiology and antimicrobial resistance.

## Methods

### Fecal samples

Fifty-six fecal samples were collected between January 2015 and December 2017 from inpatients admitted to a tertiary referral pediatric hospital in Fortaleza, Brazil.

The study included young individuals aged over 18 months and under 18 years old who were admitted to the ward or a specialized outpatient clinic with diarrhea, defined as the presence of three liquid stools over 24 hours were considered for inclusion.

Stool samples were collected from nosocomial or community-acquired diarrhea patients. Only two infants younger than 18 months were included in this study: one for having Hirschspring's disease, a possible risk factor for developing severe *C. difficile* infections, and the other for sharing a ward with two children diagnosed with CDI. This infant was included based on literature showing that *C. difficile* spores can persist for long periods in healthcare environments and are resistant to most disinfectants and cleaning agents.

### C. difficile toxin detection

All samples were collected during episodes of active diarrhea, and each sample was subsequently tested for toxins A/B using the ProSpecT *C. difficile* Toxin A/B Microplate (Remel®), following the manufacturer's instructions. This test has exhibited excellent sensitivity and specificity.^8^

### C. difficile isolation

Stool samples were also cultured using the standardized process of isolation and identification.^9^ Briefly, alcohol shock was performed on the stool sample, followed by culture on Cefoxitin and Cycloserine and Fructose Agar (CCFA, Oxoid®) and Fastidious Anaerobe Broth (FAB) for 5 and 15 days, respectively, incubated under anaerobic conditions (anaerobic jar 90 % N_2_, 10 % CO_2_). Then, the FAB was inoculated onto another CCFA. Characteristic *C. difficile* colonies on CCFA appeared yellowish, with a ground-glass appearance; they were circular with slightly filamentous edges and flat to low with a rounded elevation. The colonies were lipase- and lecithinase-negative using an Egg-Yolk Agar (EYA). These colonies were inoculated onto Brucella agar with 5 % lysed sheep blood and vitamin K (5 mg/mL) for identification and molecular tests. Identification was confirmed through testing with the RapID ANA II system (Remel®) and by amplifying the *tpi* gene by PCR.

### The toxigenic profile of the isolates

Genomic DNA from each strain was obtained from overnight cultures in Brain Heart Infusion broth (BHI; Oxoid®) using the InstaGeneTM reagent (BioRad®). Fragments of *tcdA, tcdB, cdtB*, and *tcdC* were amplified by PCR, utilizing established primers and conditions.^9^

### PCR-ribotyping

Intergenic spacer regions were amplified for ribotyping using Bidet primers as described previously.^10^ PCR-ribotypes were determined by submitting data to the web database WEBRIBO (http://webribo.ages.at).

PCR ribotyping was analyzed by the Laboratory of Biology of Anaerobes of the Medical Microbiology Department ‒ IMPG at the Federal University of Rio de Janeiro (UFRJ).

### PFGE typing

The PFGE procedure employed in this study was adapted from established protocols.^9^ Subsequently, the obtained images were analyzed using BioNumerics software (version 5.1, Applied Maths), and the resulting macrorestriction patterns were compared to those stored in the databases of the National Microbiology Laboratory of the Public Health Agency of Canada, located in Winnipeg, Canada and the University of Costa Rica.

### Antimicrobial susceptibility testing

Minimum Inhibitory Concentrations (MIC) for clindamycin, levofloxacin, moxifloxacin, rifampicin, metronidazole, and vancomycin were determined using E-test® (bioMérieux®).^11^ Resistance breakpoints were established following the Clinical and Laboratory Standards Institute (CLSI) guidelines (M11-A9) as follows: clindamycin ≥ 8 mg/mL; moxifloxacin ≥ 4 mg/mL; rifampin ≥32 mg/mL, metronidazole ≥ 32 mg/mL and vancomycin > 2 mg/mL.

We used the moxifloxacin breakpoint for both ciprofloxacin and levofloxacin. In the case of vancomycin, we adhered to the guidelines provided by EUCAST (European Committee on Antimicrobial Susceptibility Testing), accessible at http://www.eucast.org/clinical_breakpoints/. As for rifampicin, we utilized the breakpoint previously established.^12^

### Statistical analyses

The data are presented as means ± Standard Error (SEM) or as medians when applicable. Descriptive analysis of patient characteristics was conducted using univariate analysis and the Chi-Square test; p-values of < 0.05 were considered statistically significant. All analyses were carried out using IBM SPSS® Statistics 20 software.

## Results

A total of 56 specimens were evaluated. Diarrheic stool samples were collected from 49 children; five patients contributed with more than one sample, collected at different points during their hospitalization. Detailed information regarding patients' demographic, socio-economic, and health characteristics is provided in Table 1.

**Table 1:** Information on patients' demographic, socio-economic, and health characteristics in Tertiary Pediatric Hospital, Fortaleza, Brazil (2015–2017).

**Variables**	***C. difficile infection***	***Non-C.difficile infection***		**Multinomial Logistic Regression**			
	**n (%)**	**n (%)**	**p-value**	**p-value**	**Wald**	**OR**	**IC**
**Age (yeas)**^a^	8.0 (±5.40)	11.0 (±5.10)	0.086				
**Gender**^b^							
Male	9 (52.9)	19 (48.7)	0.771				
Female	8 (47.1)	20 (51.3)					
**Residence**^b^							
Urban	9 (52.9)	23 (59.0)	0.772				
Rural	8 (47.1)	16 (41.0)					
**Clinical symptoms:**							
**Diarrhea**^b^	17 (100.0)	39 (100.0)	1.000				
Length of diarrhea (days)^a^	11 (±12)	23 (±41)	0.234				
Presence of mucos^b^	7 (41.2)	19 (48.7)	0.772				
Presence of blood^b^	7 (41.2)	15 (38.5)	0.848				
**Nausea**^b^	9 (52.9)	9 (23.1)	**0.035**	**0.032**	4.591	1.119	12.564
**Vomit**^b^	9 (52.9)	11 (35.9)	0.128				
**Fever**^b^	7 (41.2)	21 (53.8)	0.562				
**Abdominal pain**^b^	14 (82.4)	27 (69.2)	0.351				
**Abdominal distension**^b^	3 (17.6)	11 (28.2)	0.513				

n (%), number (percentage); CI, confidence interval; OR, odds ratio.

a Mann-Whitney Test.

b Chi-square Test (χ²). Significant value in bold when *p* < 0.05.

**Fig. 1:**
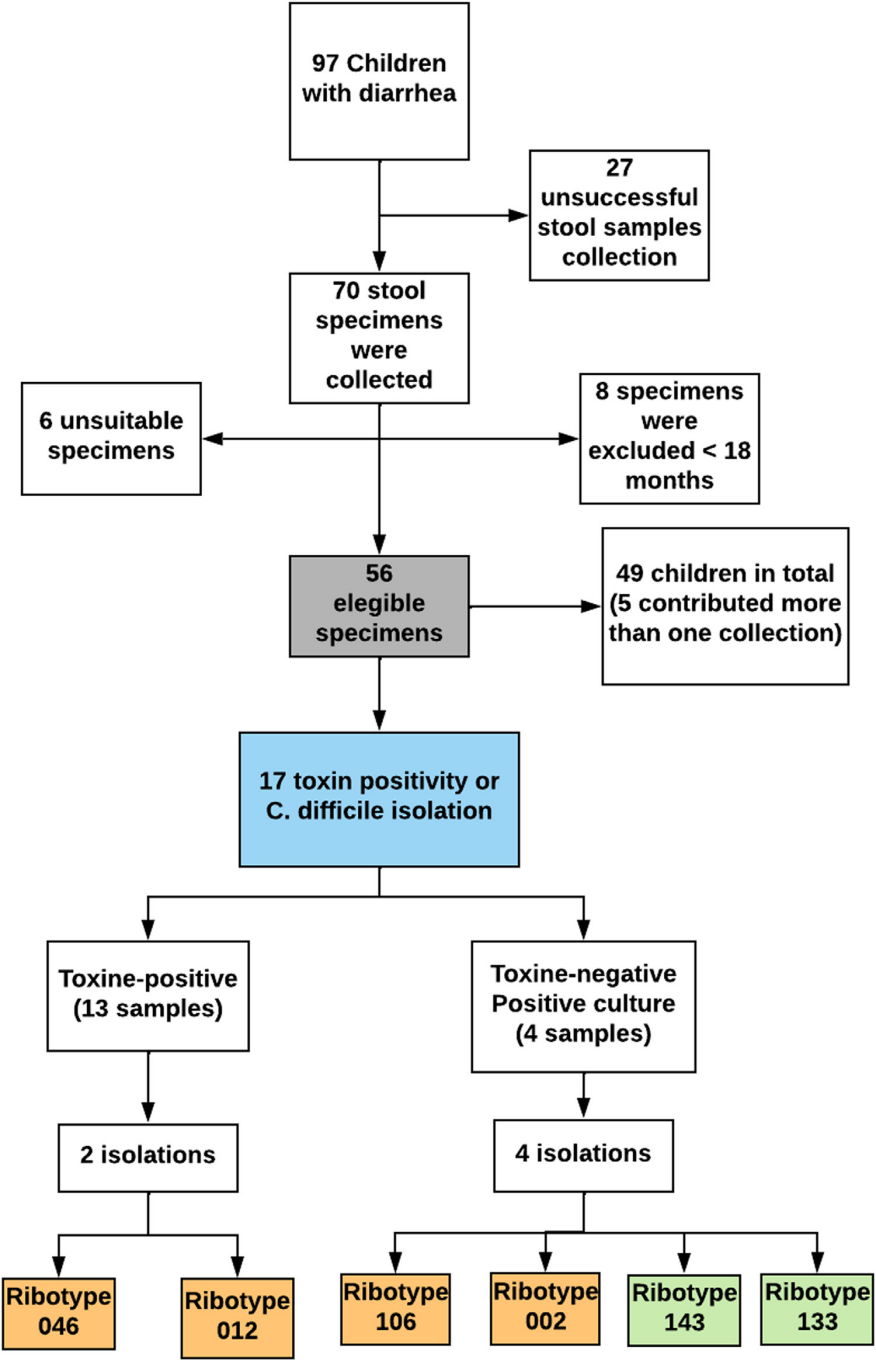
Flow Chart indicating the recruitment and isolation of *Clostridioides difficile* in children with diarrhea at a tertiary hospital in Fortaleza, Brazil, between 2015 and 2017. Toxigenic ribotypes are highlighted in orange, while non-toxigenic ribotypes are highlighted in green.

In this study, the frequency of *C. difficile* positivity (toxins and/or isolation) was 30.4 % (17/56). Initially, *C. difficile* toxins were detected in 13 of 17 samples through toxin A/B rapid immunoassay (76.4 %), among which two isolates were obtained. Four isolates were obtained from the toxin-negative samples, of which two were subsequently tested as toxigenic through PCR. Consequently, the percentage of *C. difficile* isolated in culture was 35 % (6/17). None of the children presented more than one positive sample (Fig. 1).

According to the Laboratory of Biology of Anaerobes at UFRJ, the isolates of *C. difficile* belonged to six different ribotypes: 046, 106, 002, 143, 133, and 012 (Table 2).

**Table 2:** Characterization of the *C. difficile* strains isolated from patients hospitalized in Tertiary Pediatric Hospital, Fortaleza, Brazil (2015–2017).

**Strains**	**Ribotype**	**PCR**	**Pulsotype**	**MICs (ug/mL)**					
				**MET**	**VAN**	**CLI**	**LEV**	**MXF**	**RIM**
MRN-01	046	*tpi+, tcdA+, tcdB+*, w/o deletions *tcdC, cdtB-*	New pulsotype 1174	0.38	1.5	1	4	1	<0.002
MRN-15	106	*tpi+, tcdA+, tcdB+*, w/o deletions *tcdC, cdtB-*	NAP11 0499	0.5	2	3	4	1.5	0.002
MRN-54	002	*tpi+, tcdA+, tcdB+*, w/o deletions *tcdC, cdtB-*	New pulsotype NML-1234	0.125	0,5	4	1	0.75	<0.002
MRN-55	143	*tpi+, tcdA-, tcdB-*, w/o deletions *tcdC, cdtB-*	Non-Tox Strain						
MRN-56	133	*tpi+, tcdA-, tcdB-*, w/o deletions *tcdC, cdtB-*	Non-Tox Strain						
MRN-58	012	*tpi+, tcdA+, tcdB+*, w/o deletions *tcdC, cdtB-*	New pulsotype NML-1235	0.125	0.75	2	1	0.5	<0.002

Toxigenic profile – *tcdA* (TcdA gene), *tcdB* (TcdB gene), *cdtB* (gene for the binding domain of the binary toxin) e *tcdC* (negative regulator gene that controls the production of TcdA and TcdB). MICs, Minimum Inhibitory Concentrations; MET, Metronidazole; VAN, Vancomycin; CLI, Clindamycin; LEV, Levofloxacin; MXF, Moxifloxacin; RIM, Rifampicin; MRN, Medical Record Number. Breakpoints: levofloxacin ≥4 µg/mL; clindamycin ≥8 µg/mL; moxifloxacin ≥4 µg/mL; rifampin ≥32 µg/mL, metronidazole ≥32 µg/mL and vancomycin >2 µg/mL.

Toxin and *tpi* gene fragments (definitive identification) were performed on all the isolated strains. PCR targeting *tpi, tcdA*, and *tcdB* genes were found in Medical Record Number (MRN)-1 (RT046), MRN-15 (RT106), MRN-54 (RT002), and MRN-58 (RT012) samples, but no deletion of the *tcdC* gene was found in these samples. The samples MRN-55 (RT143) and MRN-56 (RT133) exhibited non-toxigenic characteristics, and therefore genotyping by PFGE was not performed. The binding gene of the binary toxin was not detected in any of the samples (Table 2).

The analysis of the PFGE profile by digestion with *Sma*I revealed new pulsotypes in three isolates (RT046, RT002, and RT012). In addition, RT106 was identified as NAP11-0499. The analysis of the identified number was standardized according to the database of the University of Costa Rica and NML-Canada (Table 2).

In terms of antimicrobial susceptibility, all strains were susceptible to metronidazole and vancomycin, commonly used for treating CDI. The children with CDI, including those six patients whose stool samples tested positive for *C. difficile*, were treated with metronidazole and evolved with diarrhea remission during the follow-up period. All strains were susceptible to the tested antimicrobials except two that showed levofloxacin resistance (MRN-1 and MRN-15), as shown in Table 2.

Regarding clinical manifestations, shown in Table 1, most affected children experienced acute diarrhea, typically lasting up to 14 days. However, it's worth noting that three patients experienced persistent diarrhea lasting between 14 and 30 days, while two children had chronic diarrhea lasting more than 30 days. Alongside diarrhea, additional symptoms such as vomiting, fever, abdominal pain, and abdominal distension were noted. However, only nausea exhibited a significant association (*p* = 0.035) with patients who tested positive for CDI in comparison to those with a negative *C. difficile* infection test (Table 1).

When evaluating risk factors for CDI, which include recent hospitalization (within the last 30 days), antibiotic or proton pump inhibitor usage, and the presence of comorbidities or chronic diseases, it was observed that 88.2 % (15/17) of patients with positive samples had underlying conditions. Importantly, each of these 15 children presented one or more risk factors. Only two children with positivity to *C. difficile*, constituting 11.7 % (2/17), exhibited no risk factors (Table 3). Hospitalization within the last 30 days was noted in 41.1 % (7/17) of patients with positivity to *C. difficile*, with no significant difference compared to those with negative *C. difficile* test (*p* = 0.206). Antibiotics had been previously prescribed to 47 % (8/17) of children with CDI, and proton pump inhibitors had also been once used by 47 % (8/17) of these children (Table 3).

**Table 3:** Clinical correlations of patients with *C. difficile* isolation in a Tertiary Pediatric Hospital, Fortaleza, Ceará (2015–2017).

**Characteristics**	**Patients**					
	**MRN-01**	**MRN-15**	**MRN-54**	**MRN-55**	**MRN-56**	**MRN-58**
Ribotype	046	106	002	143	133	012
Sex	F	M	M	M	F	M
Age	2y5m	1y5m	1y1m	13y	4y	2y5m
Diagnostic	Bile duct atresia/ transplant	Congenital megacolon	Hemolytic uremic syndrome	Inflammatory bowel disease	Chronic kidney disease	Chronic kidney disease
Risk factors						
Comorbidities	Yes	Yes	Yes	Yes	Yes	Yes
Use of antibiotics	No	Yes	Yes	No	No	Yes
Use of PPI	No	Yes	Yes	No	Yes	No
Hospitalization in the last 30 days	No	Yes	No	No	Yes	Yes
Clinical condition						
Fever	No	Yes	Yes	No	No	No
Blood in the stool	Yes	No	No	No	Yes	No
Nausea/Vomit	No	Yes	Yes	Yes	Yes	Yes
Abdominal pain	Yes	Yes	Yes	Yes	Yes	Yes

F, female; M, male; PPI, proton pump inhibitors.

While comorbidities emerged as the most prevalent risk factor, affecting 76.5 % (13/17) of CDI patients, no statistical differences were found between patients with and without the infection (*p* = 0.758). These comorbidities included inflammatory bowel disease (35.3 %; 6/17), chronic kidney disease (23.5 %; 4/17), neurologic disease (11.8 %; 2/17), and hepatopathies (11.8 %; 1/17), as shown in Table 3. However, it is crucial to emphasize that all six patients infected with *C. difficile*, from whom fecal isolates were obtained, had previously been diagnosed with a chronic disease.

## Discussion

Considering the changing patterns of *C. difficile* infection in childhood,^7^^,^^13^, ^14^, ^15^, ^16^, ^17^, ^18^, ^19^ the present study investigated the molecular epidemiology and antimicrobial resistance in *C. difficile* strains isolated from children and adolescents in a tertiary referral pediatric hospital in Fortaleza, Brazil, and the associated risk factors. As far as we know, no previous research has examined *C. difficile* infection among children in northeastern Brazil. Studies on *C. difficile*-associated diarrhea in Brazil have been limited. In the last two decades, only two pediatric research groups reported the presence of *C. difficile* infection in Brazilian children: a study carried out in Rio de Janeiro with hospitalized and non-hospitalized patients^20^ and a study conducted in São Paulo between June 2000 and July 2001.^21^ In these studies, *C. difficile* was isolated from 6.7 % and 5.5 % of diarrheal stool samples, respectively. In this current study, the frequency of *C. difficile* positivity stood notably much higher at 30.4 % (17/56), with the rate of toxigenic *C. difficile* reaching 88.2 %, detected by toxin A/B rapid immunoassay and PCR, accounting for 15 out of 17 cases.

Further epidemiological studies are needed to confirm whether pediatric CDI has increased in Brazil, but our data, compared with studies from Brazilian hospitals in the 2000s, point in that direction. This hypothesis aligns with studies by other research groups in different countries.^22^^,^^23^

This difference between the Brazilian studies conducted in the 2000s and our study can be attributed to a combination of factors. During this time gap, changes in health conditions, hygiene practices, antibiotic use, emergence of new strains, and other factors that affect the prevalence of *C. difficile* and other intestinal pathogens may have occurred. Furthermore, in Brazil's case, it's crucial to consider the vast continental proportions of the country, which are geopolitically divided into five regions. For instance, Fortaleza is the capital of the state of Ceará, situated in the northeastern region, while Rio de Janeiro and São Paulo are in southeastern Brazil's wealthiest region. Tailored epidemiological studies in each region are essential to gather relevant, region-specific data on *Clostridioides difficile*-associated diarrhea. In this context, our study stands out as a significant contribution, as it provides insights into the local epidemiological landscape of CDI among children in northeastern Brazil for the first time.

We should also consider the age group differences among these Brazilian studies as a variable that may affect the results: Rio de Janeiro (3 months ‒ 7 years), São Paulo (0‒5 years), and Fortaleza (18 months ‒ 18 years). In this study, we excluded children under 18 months old due to elevated asymptomatic carriage rates among infants and young children. This significantly complicates diagnosing *C. difficile*-associated disease in this group, leading to challenges in defining infection and determining the necessity of treatment.^23^ Two exceptions were made for infants under 18 months. One had Hirschsprung's disease, a potential risk factor for severe CDI. The other shared a ward with two children already diagnosed with CDI, heightening the risk of infection transmission due to the persistent presence of *C. difficile* spores in healthcare environments. Indeed, both infants were confirmed to be infected with non-toxigenic C. difficile strains (ribotypes 106 and 002).

We also examined commonly reported CDI risk factors, which include hospitalization, previous antibiotics exposure, previous use of Proton Pump Inhibitors (PPIs), and comorbidities. Overall, 88.2 % (15/17) of the children infected with *C. difficile* were identified as having at least one of these risk factors. Comorbidity was the most prevalent risk factor 76.5 % (13/17), followed by previous use of antibiotics at 47 % (8/17) and PPIs at PIs 47 % (8/17). However, it's important to note that none of these risk factors were found to predispose to CDI in the present study. Although the above-listed factors have been commonly described for CDI in adults, it has been argued that not all CDI risk factors act equally in pediatric and adult patients.^24^^,^^25^

A case-control study to identify potential risk factors for CDI at a pediatric quaternary care hospital in the USA reported that recent antibiotic exposure and certain comorbid conditions (solid organ transplant, presence of a gastrostomy, or jejunostomy tube) were associated with CDI.^26^ One of the toxigenic strains identified in the present study (ribotype 046 with a new pulsotype 1174) came from a child with a history of hospitalization, antibiotic administration, hepatic transplantation, and immunosuppressive medication therapy. In addition to ribotype 046, we identified five more *C. difficile* ribotypes (106, 002, 143, 133, 012). Among these, three new pulsotypes were detected (1174, NML-1234, NML-1235) and NAP 11–0499.

The heterogeneity found in the PCR-ribotyping of *C. difficile* strains performed in the present study does not suggest an outbreak. In a study conducted in eight US states to examine the association between strain type and disease outcomes in adults, NAP11 was previously identified as the third most common strain type (9.1 %).^26^ A study conducted in Chicago, USA, with pediatric patients diagnosed with CDI reported the evolution and transmission of a multi-drug resistant DH/NAP11/106 *C. difficile* clone.^27^ In accordance, NAP11/RT106, isolated in our study, was resistant to levofloxacin. Fluoroquinolone resistance in a BI/NAP1/027 strain has been implicated as a significant contributor to its pathogenesis and global spread.^28^^,^^29^ All *C. difficile* strains isolated in the present study were sensitive to metronidazole and vancomycin, the most used antibiotics to treat CDI in Brazil. Therefore, all children responded well to metronidazole treatment.

Our study obtained four isolates from samples that initially tested negative for toxins. Based on a careful evaluation of the clinical features and associated risk factors, we decided to treat these four patients with metronidazole, as there were strong indications that they might be infected with *C. difficile*. Subsequently, two toxigenic strains were isolated from this group, emphasizing the need to revise existing clinical protocols for diagnosing and treating CDI, especially in the pediatric population.

## Conclusions

Our study provides insights into the current epidemiological status of *Clostridioides Difficile*-associated Diarrhea (CDI) among children in northeastern Brazil. It reveals a substantial CDI frequency of 30.4 %, with toxigenic strains detected in 76.4 % of cases, highlighting a higher prevalence compared to earlier Brazilian studies. While our study provides valuable insights, it is essential to acknowledge its limitations, notably the small sample size and restriction to a single pediatric hospital. These constraints underline the necessity for more extensive, multicenter studies across diverse healthcare settings in Brazil.

## Ethics approval and consent to participate

This study received approval from the Research Ethics Committee (REC) of Albert Sabin Children's Hospital (protocol number 861363). Written informed consent was obtained from all patients. The REC's primary responsibility is safeguarding the dignity, rights, safety, and well-being of individuals participating in biomedical research. It also upholds public accountability by publishing its decisions. All institutions involved in this research provided their consent to participate.

## Consent for publication

Not applicable.

## Availability of data and materials

The data generated and analyzed during the current study are available from the corresponding author upon reasonable request.

## Authors' contributions

All authors contributed to the study conception, design, and experiment. GACB and RFCL contributed equally to this work. HBRB, CLC, EOF, and DMP prepared material and collected data. HBRB, GACB, RFCL, and CQG analyzed the data; HBRB, CLC, RFCL, EOF, and GACB wrote the first draft of the manuscript. CLC, RFCL, and GACB did the final review. All authors read and approved the final manuscript.

## Funding

This work was supported by PRONEX/FUNCAP/CNPq of Brazil through grant PR2-0101-00060.01.00/15.

## Conflicts of interest

The authors declare that the research was conducted in the absence of any commercial or financial relationships that could be construed as a potential conflict of interest.
